# Efficacy of Spinal Cord Stimulation for Failed Back Surgery Syndrome in Elderly Patients: A Retrospective Study

**DOI:** 10.1155/2023/2136562

**Published:** 2023-05-09

**Authors:** Naoki Higashiyama, Shinya Tamura, Taku Sugawara

**Affiliations:** Department of Spinal Surgery, The Akita Cerebrospinal and Cardiovascular Center, Akita, Japan

## Abstract

**Objectives:**

Failed back surgery syndrome (FBSS) refers to a condition where symptoms such as low back pain, leg pain, and numbness persist or recur after lumbar surgery; it has been reported to occur in 10%–40% of patients who have undergone lumbar surgery. Spinal cord stimulation (SCS) has been reported useful for low back and leg pain due to FBSS. In this study, we studied the efficacy and safety of SCS for FBSS in older adults.

**Methods:**

Among FBSS patients who underwent an SCS trial between November 2017 and December 2020, those with at least 50% pain reduction during the trial phase who requested spinal cord stimulator implantation underwent implantation of a stimulator under local anesthesia. The patients were divided into two groups: patients aged <75 years (<75-year-old group) and patients aged ≥75 years (≥75-year-old group). The male/female ratio, symptom duration, operative duration, visual analog scale (VAS) scores before and after one year of surgery, responder rate (RR), complications one year after surgery, and stimulator removal rate were analyzed.

**Results:**

There were 27 cases in the <75-year-old group and 46 in the ≥75-year-old group, with no significant differences in male/female ratio, duration of pain, or operative time between the two groups. VAS scores for low back pain, leg pain, and overall pain one year after surgery were improved significantly from respective preoperative scores in both groups (*P* < 0.001). There were no significant differences in low back pain VAS, leg pain VAS, overall pain VAS, RR, complications one year after surgery, or stimulator removal rate between the two groups.

**Conclusion:**

SCS reduced pain effectively in both <75-year-old and ≥75-year-old groups with no differences in complications. Therefore, spinal cord stimulator implantation was considered a viable option for FBSS treatment in older adults because it can be performed under local anesthesia and is associated with a low incidence of complications.

## 1. Introduction

FBSS, a condition where symptoms such as low back pain, leg pain, and numbness persist or recur, has been reported to occur in 10%–40% of the patients who had lumbar surgery [1]. Conventional treatments for FBSS include drug therapy, physical therapy, cognitive behavioural therapy, conservative treatments such as nerve block, or repeated surgery such as lumbar decompression and fusion, but they do not produce satisfactory effects [2–6].

SCS is widely used for treating chronic intractable pain [7], and randomized controlled trials (RCTs) comparing SCS with conservative treatment and repeated surgery for low back pain and leg pain due to FBSS have shown the advantage of SCS [8–10]. While conventional paresthesia-based SCS with low-frequency stimulation is still commonly used, recently introduced stimulation methods, such as burst SCS [11, 12], 10 kHz high-frequency therapy [13, 14], high-dose SCS [15, 16], high-density SCS [17, 18], closed-loop SCS [19, 20], and differential target multiplexed programming [21, 22], have been reported to be equally or more effective than paresthesia-based SCS. Also, spinal cord stimulator implantation using anatomical placement in which electrodes are placed mainly in the T9/10 intervertebral space has comparable therapeutic efficacy with paresthesia-based SCS but shorter operative time [23].

In this study, we compared outcomes of percutaneous SCS implantation, a minimally invasive procedure under local anesthesia, between older patients with FBSS aged <75 years and those aged ≥75 years to clarify the efficacy and safety. The primary outcome was the comparison of VAS scores for low back pain, leg pain, and overall pain before and one year after surgery and RR (percentage of patients whose pain decreased by ≥50%) between the <75-year-old and ≥75-year-old groups of patients who underwent spinal cord stimulator implantation.

## 2. Methods

It was a single-center retrospective study in FBSS patients who underwent permanent spinal cord stimulator implantation between November 2017 and December 2020 at the Akita Cerebrospinal and Cardiovascular Center. This study was conducted with approval from the ethical review board of the Akita Cerebrospinal and Cardiovascular Center (Akita, Japan). The ethical review board registration number for the study is 22-21. It has been registered in the UMIN Clinical Trials Registry (registration number: UMIN000050979).

### 2.1. Subject Selection

Eligible patients were older than 18 years, diagnosed with FBSS, had low back pain or leg pain persisting for at least three months, were nonresponsive to drug therapy, physical therapy, and conservative treatment such as epidural block injection, and had a 100-mm visual analog scale (VAS) score of at least 50 mm for low back or leg pain. The interval between the last block injection and SCS implantation in the included patients is at least one month. Before the trial, all subjects underwent the Hasegawa dementia scale-revised (HDS-R) and Minnesota Multiphasic Personality Inventory (MMPI) administered by a clinical psychologist. The HDS-R features a scoring system that ranges up to 30, whereby any score below 20 is indicative of suspicion of dementia. Additionally, ten clinical scales of the MMPI were assessed, with specific attention given to the detection of elevated levels of depression as manifested on the second scale. After patients with severe cognitive impairment or severe depression were excluded, only those psychologically appropriate for the treatment underwent an SCS trial. The patients who responded to a 7-day percutaneous trial with at least 50% pain reduction and requested stimulator implantation underwent permanent spinal cord stimulator implantation.

### 2.2. Implantation Method

**Figure 1:**
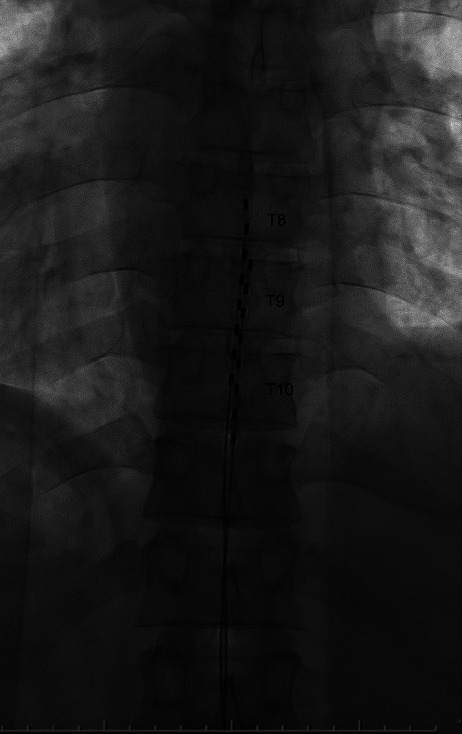
The first lead tip was placed at the midbody of T8, and the second lead tip was placed at the superior endplate of T9.

A 2-cm skin incision was made around the medial side of the left L3 pedicle as the center under local anesthesia with 1% lidocaine combined with dexmedetomidine. The dexmedetomidine is a loading dose of 6.0 *μ*g/kg administered over a period of 10 minutes, followed by a continuous maintenance infusion of 0.2 to 0.7 *μ*g/kg/hour. However, the dose was individually determined based on the patient's clinical response and potential adverse event. Tuohy needles were inserted under fluoroscopic guidance until the tip reached the epidural space via the L1/2 interlaminar foramen, and two leads (Vectris™ SureScan MRI 1 × 8 Compact, Medtronic Inc., Minneapolis, MN, USA) were then inserted. The leads were placed using an anatomical placement technique without paresthesia mapping. The first lead was placed median, and the electrode at the most cranial position was located in the center of the T8. The second lead was placed paramedian, and the electrode at the most cranial position was positioned at the cranial end of the T9 (Figure 1). Tuohy needles were removed, and the leads were anchored to the lumbodorsal fascia using mechanical anchors.

**Figure 2:**
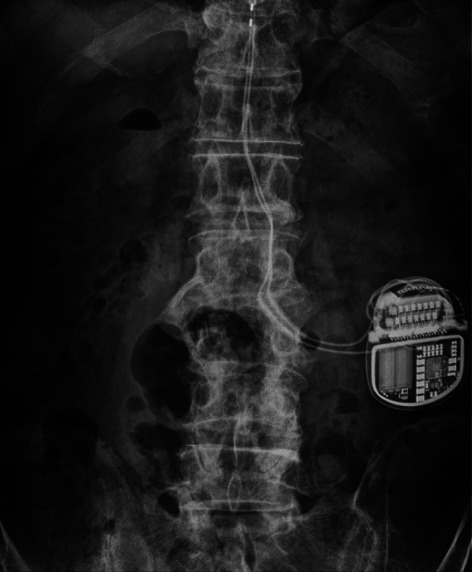
The implantable rechargeable neurostimulator was implanted between the 12th rib and the iliac crest.

We planned to place an implantable pulse generator (IPG) (INTELLIS™, Medtronic Inc., Minneapolis, MN, USA) between the low end of the costae and the iliac crest or in the gluteal region (Figure 2). A 5-cm transverse incision was made to prepare a subcutaneous pocket. The leads guided subcutaneously were connected to the IPG, and lead resistance values were checked. Additional strain relief loops were created in the IPG pocket before connecting the leads. The purpose of strain relief loops is to prevent tension or pulling on the lead from damaging the connection or causing the lead migration. The leads were placed in the subcutaneous pocket, and the wound was closed. Immediately after surgery, therapy was initiated utilizing a bipolar electrode directed towards the T9/10 intervertebral space. All participants were programmed to receive a high-dose SCS protocol featuring a pulse width of 90 *μ*sec, a rate of 1 kHz, and a subperception threshold amplitude to enable continuous stimulation. The subperception threshold amplitude was defined as 70–80% of the perception threshold, which was determined by gradually increasing the amplitude while in the supine position until paresthesias were observed.

### 2.3. Definition of Older Adults

Adults aged ≥65 years are defined as elderly in many countries; however, clear global criteria to define older adults have not been established. In Japan, where the population is aging more rapidly than in other countries, older adults have improved physical functions in recent years, and the Japan Geriatrics Society has proposed to define adults aged ≥75 years as being elderly [24]. Therefore, in this study, individuals aged ≥75 years were defined as being elderly, and comparisons were made between patients aged <75 years (the <75-year-old group) and those aged ≥75 years (the ≥75-year-old group).

### 2.4. Parameters Analyzed

The following parameters were compared between the <75-year-old and ≥75-year-old groups of patients who underwent spinal cord stimulator implantation: male/female ratio, symptom duration (since diagnosis), the operative time for permanent implantation, VAS scores for low back pain, leg pain, and overall pain before and one year after surgery, RR (percentage of patients whose pain decreased by ≥50%), complications at one year after surgery, and stimulator removal rate. As more than half of the participants in this study were aged 75 years or older, age-related comorbidities and functional decline made it difficult to adjust for age differences in functional assessments such as the Oswestry Disability Index (ODI), Short Form (12) Health Survey (SF-12), Short Form (36) Health Survey (SF-36), and EuroQol-5 Dimension 5-Level (EQ-5D-5L). In addition, older individuals may have difficulty completing such functional assessment questionnaires due to potential cognitive impairment. Therefore, only pain intensity was reported as an outcome measure in this study.

### 2.5. Statistical Analysis

Fisher's exact test and *t*-test were used to compare categorical variables and preoperative/postoperative VAS scores. Mann–Whitney *U* test was used for intergroup comparisons of other variables. Differences with *P* < 0.05 were considered statistically significant. Kaplan–Meier curves were used to analyze the difference in explantation rates and adverse event rates over time between the <75-year-old and ≥75-year-old groups of patients who underwent spinal cord stimulator implantation. SPSS (IBM SPSS Statistics for Windows, Version 28.0.1; IBM Corp., Armonk, NY, USA) was used for data analysis.

## 3. Results

Seventy-three patients (33 men and 40 women) with a mean age of 74.8 ± 10.5 years underwent spinal cord stimulator implantation between November 2017 and December 2020. The number of patients, mean age, symptom duration, and operative duration were 27 patients (12 men and 15 women), 63.6 ± 8.2 years, 67.6 ± 93.2 months, and 55.2 ± 22.8 minutes, respectively, for the <75-year-old group, and 46 patients (21 men and 25 women), 81.3 ± 4.4 years, 66.0 ± 81.9 months, and 57.7 ± 19.2 minutes, respectively, for the ≥75-year-old group. There were no significant differences between the two groups in the male/female ratio (*P*=1.000), symptom duration (*P*=0.489), and operative duration (*P*=0.163) (Table 1).

As to treatment outcomes of the <75-year-old group, low back pain VAS scores before and one year after surgery were 78.6 ± 12.0 mm and 28.7 ± 19.9 mm, respectively, leg pain VAS scores before and one year after surgery were 76.4 ± 17.3 mm and 24.1 ± 15.8 mm, respectively, and overall pain VAS scores before and one year after surgery were 84.3 ± 10.9 mm and 30.0 ± 19.2 mm, respectively, for the ≥75-year-old group, and low back pain VAS scores before and one year after surgery were 78.0 ± 14.3 mm and 31.6 ± 20.2 mm, respectively, leg pain VAS scores before one year after surgery were 77.0 ± 14.2 mm and 24.1 ± 18.6 mm, respectively, and overall pain VAS scores before and one year after surgery were 85.8 ± 8.6 mm and 32.5 ± 20.9 mm, respectively. VAS scores for low back pain, leg pain, and overall pain one year after surgery were improved significantly from respective preoperative scores in both groups (*P* < 0.001). One year after surgery, the RRs for low back, leg, and overall pains were 81.5%,81.5%, and 81.5% for the <75-year-old group and 80.4%, 82.6%, and 82.6% for the ≥75-year-old group. For the primary outcome measure, there were no significant differences between the <75-year-old group and the ≥75-year-old group in low back pain VAS scores before and one year after surgery (*P* = 0.936 and 0.391), leg pain VAS scores before and one year after surgery (*P*=0.995 and=0.598), overall pain VAS scores before and one year after surgery (*P*=0.775 and *P*=0.511), and RRs (all *P*=1.000) (Table 2).

**Figure 3:**
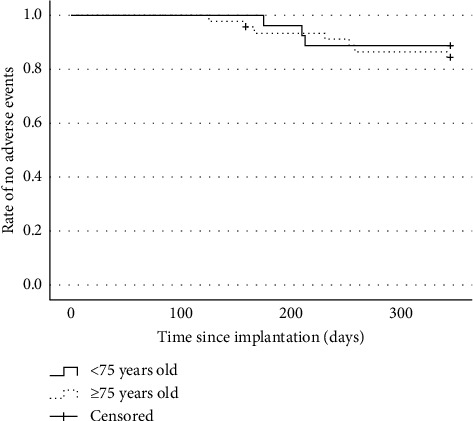
Kaplan–Meier survival curve for the time to onset of adverse events (log-rank *P*=0.617).

**Figure 4:**
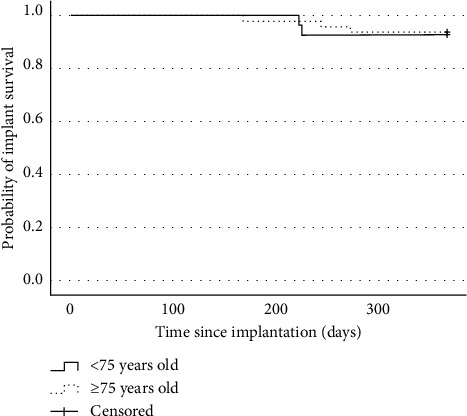
Kaplan–Meier survival curve for the time to explantation (log-rank *P*=0.875).

The following complications occurred within one year after surgery (cases in the <75-year-old group versus the ≥75-year-old group): lead migration (1 versus 3, *P*=1.000), implant-related pain (1 versus 3, *P*=1.000), infection (0 versus 1, *P*=1.000), and metal allergy (1 versus 0, *P*=0.370). No cerebrospinal fluid leaks and nerve injuries occurred. Implant-related pain was defined as pain associated with the component site of the device, such as pain around the IPG site or at the lead anchor site. Within one year after surgery, a total of five patients, two (7.4%) in the <75-year-old group and three (6.5%) in the ≥75-year-old group, underwent stimulator removal for implant-related pain (2 cases), infection (1 case), metal allergy (1 case), and loss of therapeutic effect (1 case). The complications and stimulator removal incidence rates did not differ significantly between the two groups (*P*=1.000) (Table 3). Kaplan–Meier curves showed a nonsignificant difference in adverse event rates (log-rank *P*=0.617) (Figure 3) and explantation rates (log-rank *P*=0.875) (Figure 4) between the <75-year-old and ≥75-year-old groups of patients who underwent spinal cord stimulator implantation.

## 4. Discussion

The evidence available for the effectiveness of conventional drug therapy and repeated surgery for FBSS is weak [10]. On the other hand, SCS has been shown to alleviate pain more effectively than conservative treatment and repeated surgery [8–10] and has proven to be a safe and effective treatment strategy [25]. In this study, we also showed significant improvements in the low back and leg pain VAS scores one year after surgery.

The <75-year-old and ≥75-year-old groups in this study did not differ significantly in the male/female ratio, symptom duration, and operative duration. This study also showed no age-dependent differences in the treatment outcomes measured as VAS and RR. The data available in the literature about SCS outcomes in patients aged ≥75 years are limited, and there is a paucity of reports on whether age has effects on treatment outcomes. The issue remains controversial because studies report the absence of age-dependent differences in treatment outcomes [26–28], those reporting that the RR in pain relief decreased with age [29, 30], and those reporting that pain was removed more effectively in older patients [31, 32]. A recent retrospective study by Bondoc et al. in 189 patients has shown negative correlations of age with sensory and affective components of pain, suggesting that these pain components may be improved more effectively in older patients [32].

In recent studies using the stimulator removal rate to evaluate outcomes in patients who underwent SCS, the explant rate ranged from 5.96% to 11.6% [28, 33–37]. As to whether age affects the incidence of stimulator removal, some studies have reported that age has no effects [28, 36], while others have reported that the incidence was higher in younger patients [33–35]. Hussain et al. have reported that 3104 of 52070 patients (5.96%) underwent removal of stimulators within two years after implantation, 72.8% of them did so in the first year, and the odds ratio for removal was lower in older patients [37]. The stimulator removal rates in the present study were comparable with previously reported rates and did not differ depending on age. Aside from age, factors reported to have effects on the stimulator removal rate include smoking [35, 37], depression [34, 37], psychiatric diseases [35], radiculopathy [36], female sex [34, 37], falls after implantation [28], and device manufacturer [28]. In recent studies in large samples, older patients had lower stimulator removal rates and better treatment outcomes; however, a meta-analysis of age-associated changes in pain has revealed that the pain threshold increases with age, and older adults have a decreased sensitivity to low-intensity pain [38], suggesting an association.

As to complications, lead migration is generally most common (15%), followed by lead fracture, malfunction, implant-related pain, and infection (5%–6%) [39]. Percutaneous SCS implantation has also been reported to be associated with lower rates of stimulator removal due to infection and reintervention due to medical-related complications compared with SCS implantation via laminectomy [40]. The use of a small incision technique and anatomical placement in this study to decrease the invasiveness and duration of surgery may have contributed to a lower incidence of infection than previously reported rates.

No global consensus about age to define older adults has been established. In this study, the <75-year-old group had a mean age of 63.6 years old and included many subjects who would be classified as older in other studies using a lower age to define older adults. This may explain why there were no differences in treatment outcomes and the device removal rate.

The diagnostic label “failed back surgery syndrome” may be considered unsatisfactory, inaccurate, and potentially problematic by medical professionals managing patients with persistent pain after spine surgery. The International Association for the Study of Pain has recommended the use of the term “persistent spinal pain syndrome (PSPS) type 2” as a more appropriate diagnostic label than FBSS. The term PSPS encompasses the various potential symptoms of a syndrome of chronic pain (according to the usual criteria for determining pain persistence) or recurrent pain of spinal origin, paresthesias, numbness, stiffness, muscle spasms and weakness, and in some cases sphincter dysfunction [41].

Limitations of this study are the absence of functional outcome data, its retrospective nature, the small sample size, and the inclusion of a single center. Functional outcome is a crucial point in the treatment of patients with chronic pain as functional improvement is as important as pain reduction. It was evaluated 12 months after SCS implantation, and it would be of interest to follow patients over a longer period. Moving forward, it is desirable to conduct a prospective multicenter study on the relationships between age and SCS outcomes, along with other potential predictors.

## 5. Conclusion

In this study, the treatment outcomes and complications after SCS implantation did not differ between the <75-year-old group and the ≥75-year-old group. Since spinal cord stimulator implantation can be performed under local anesthesia and is associated with a low incidence of complications, therefore, SCS can be a highly effective treatment option for FBSS in older adults.

## Figures and Tables

**Table 1:** Subject demographics.

	<75 years old *n* = 27	≥75 years old *n* = 46	*P* value
Age mean (SD)	63.6 (8.2)	81.3 (4.4)	<0.001^†^
Gender *n*			
Female	15	25	1.000^*∗*^
Male	12	21	
Duration of pain (months) mean (SD)	67.6 (93.2)	66.0 (81.9)	0.489^†^
Operative time (min) mean (SD)	55.2 (22.8)	57.7 (19.2)	0.163^†^
Previous spine surgery type			
Laminectomy	22	38	1.000^*∗*^
Fusion	2	6	0.702^*∗*^
Discectomy	3	2	0.352^*∗*^
Level of operation			
L3/4 and above	4	5	0.718^*∗*^
L4/5	12	24	0.630^*∗*^
L5/S1	3	4	0.705^*∗*^
Multiple levels	8	13	1.000^*∗*^
Number of previous spine surgeries mean (SD)	1.6 (1.1)	1.3 (0.7)	0.392^†^

^*∗*^Fisher's exact test, ^†^Mann–Whitney *U* test.

**Table 2:** VAS scores and responder rate between baseline and 1-year postoperative follow-up. A responder was defined as achieving at least a 50% improvement in pain compared to baseline VAS scores.

	<75 years old *n* = 27	≥75 years old *n* = 46	*P* value
VAS mm (SD)			
Low back pain			
Baseline	78.6 (12.0)	78.0 (14.3)	0.936^†^
1 year	28.7 (19.9)	31.6 (20.2)	0.391^†^
Leg pain			
Baseline	76.4 (17.3)	77.0 (14.2)	0.995^†^
1 year	24.1 (15.8)	24.1 (18.6)	0.598^†^
Overall pain			
Baseline	84.3 (10.9)	85.8 (8.6)	0.775^†^
1 year	30.0 (19.2)	32.5 (20.9)	0.511^†^
Responder rate			
Low back pain	81.5%	80.4%	1.000^*∗*^
Leg pain	81.5%	82.6%	1.000^*∗*^
Overall pain	81.5%	82.6%	1.000^*∗*^

^*∗*^Fisher's exact test, ^†^Mann–Whitney *U* test.

**Table 3:** Adverse events and explant. Reasons for spinal cord stimulator explanation and adverse events in this study.

	<75 years old *n* = 27	≥75 years old *n* = 46	*P* value^*∗*^
Adverse events *n* (%)	3 (11.1%)	7 (15.2%)	0.735
Lead migration	1	3	1.000
Implant-related pain	1	3	1.000
Infection	0	1	1.000
Metal allergy	1	0	0.370
Incidence of explant *n* (%)	2 (7.4%)	3 (6.5%)	1.000
Implant-related pain	1	1	1.000
Infection	0	1	1.000
Metal allergy	1	0	0.370
Loss of efficacy	0	1	1.000

^*∗*^Fisher's exact test.

## Data Availability

The data supporting the current study are available from the corresponding author upon request.
